# Neoadjuvant chemotherapy is associated with a high rate of perioperative blood transfusion at the time of interval cytoreductive surgery

**DOI:** 10.1186/s12885-018-4882-8

**Published:** 2018-10-26

**Authors:** Kevin W McCool, Emmanuel Sampene, Brock Polnaszek, Joseph Connor, Erin E Medlin, Lisa Barroilhet

**Affiliations:** 1 Division of Gynecologic Oncology, Department of Obstetrics and Gynecology, University of Wisconsin, 600 Highland Ave, Madison, WI 53792 USA; 2 Department of Biostatistics and Medical Informatics, University of Wisconsin, 600 Highland Ave, Madison, WI 53792 USA; 3 Department of Pathology and Laboratory Medicine, University of Wisconsin, 600 Highland Ave, Madison, WI 53792 USA; 4 School of Medicine and Public Health, University of Wisconsin Hospital and Clinics, University of Wisconsin, 600 Highland Ave, Madison, WI 53792 USA; 5 Penn Medicine/Lancaster General Health, LG Health Physicians Gynecologic Oncology Specialists, 2102 Harrisburg Pike, Lancaster, PA 17601 USA; 6 University of Michigan, Division of Gynecologic Oncology, Department of Obstetrics and Gynecology, 1500 E. Medical Center Drive, Ann Arbor, MI 48109-5276 USA

**Keywords:** Ovarian cancer, neoadjuvant, interval cytoreduction, blood transfusion

## Abstract

**Background:**

The oncologic safety of allogeneic blood transfusion in ovarian cancer patients is unknow. We sought to determine the prevalence and oncologic safety of perioperative allogeneic blood transfusion during interval cytoreduction surgery among women receiving neoadjuvant chemotherapy for ovarian cancer.

**Methods:**

We utilized retrospective chart review to identify a cohort of patients undergoing interval cytoreduction at a large academic tertiary referral center. We compared outcomes in patients who were exposed to perioperative blood transfusion compared with patients who were not exposed. Our primary endpoint was progression free survival; our secondary endpoint was overall survival. Baseline clinical characteristics were collected for patients in each group.

**Results:**

Sixty-six women were included in the final cohort of women undergoing interval cytoreductive surgery after NACT. A total of 51 women (77%) were exposed to allogeneic perioperative pRBC transfusion. Fifteen women (23%) were not exposed to transfusion. The baseline characteristics were generally well matched*.* Women who were not exposed to a perioperative blood transfusion were more likely to have a normalized CA125 prior to undergoing cytoreductive surgery. Preoperative hemoglobin concentration was lower in the transfusion group (10.5 g/dLvs 11.5 g/dL, *p* < 0.009). Perioperative transfusion was not associated with a significant difference in progression free survival (PFS = 7.6 months for transfused, 9.4 months for not transfused; log-rank test *p* = 0.4617). Similarly, there was no observed difference between groups for overall survival (OS = 23.6 months for transfused, 22.5 months for not transfused; log-rank test *p* = 0.1723).

**Conclusions:**

Women undergoing neoadjuvant chemotherapy for ovarian cancer are at high risk of exposure to blood transfusion at the time of interval cytoreductive surgery. Future studies will continue to evaluate the safety and impact of transfusion on ovarian cancer survival in this at risk population.

**Electronic supplementary material:**

The online version of this article (10.1186/s12885-018-4882-8) contains supplementary material, which is available to authorized users.

## Background

Blood transfusion has been scrutinized in intensive care and trauma settings, as it is consistently associated with worse outcomes [1, 2]. In cancer patients, perioperative blood transfusions have been shown to worsen survival in colorectal, breast and other malignancies [3–7]. These findings have been inconsistently replicated in patients with ovarian cancer [8, 9] but limited research in this area has been published. The treatment paradigm for patients with advanced ovarian cancer has shifted in the last eight years. The use of neoadjuvant chemotherapy (NACT) as an alternative to upfront debulking surgery has gained widespread acceptance after two randomized controlled studies showed equivalent survival between these treatment paradigms [10, 11]. We reasoned that patients undergoing going interval cytoreductive surgery after NACT would be uniquely at risk for perioperative anemia secondary to myelosuppressive chemotherapy. Consequently we anticipated a high prevalence of perioperative blood transfusion in this growing patient population. However, the impact of perioperative blood transfusion on progression-free survival (PFS) and overall survival (OS) remains an unanswered question. We identified a retrospective cohort of patients with ovarian cancer undergoing interval cytoreduction after NACT at our institution over a 3 year period to determine the prevalence of perioperative blood transfusion and explore the impact on OS and PFS.

## Methods

We utilized Electronic Health Records (EHR) from women undergoing treatment of ovarian cancer at the University of Wisconsin Hospital from January 1 2010 to December 31 2013. We selected our data collection window to coincide with the increased uptake of NACT after the publication of EORTC 55971 trial in 2010. A search of patient data from prior to 2010 showed an overall low rate of NACT at our institution. We chose to terminate data collection in 2013 to provide opportunity for maturation of survival data. We utilized ICD9 code 183 and CPT codes 58943 and 58950-58956 to identify all women undergoing surgical treatment for ovarian cancer. We then manually interrogated records to identify women who had previously received chemotherapy prior to surgical management. There are no well-defined criteria for peri-operative blood transfusion described in the literature. We chose to define the exposure of interest, perioperative allogeneic blood transfusion, as any women who received allogeneic packed red blood cells during their index hospitalization for interval cytoreduction surgery. We surmised that there is great variability in the timing and utilization of blood products depending on patient factors and provider preferences. As a result, we chose this broad definition to be maximally inclusive of exposures surrounding interval surgery. The primary outcome of interest was progression-free survival and the secondary outcome was OS, calculated as the interval from surgery until recurrence or death, respectively. Recurrence was defined as any clinically suspected recurrence by imaging, exam or biochemical findings. Demographic data including age and BMI were obtained, as well as clinical data including pre-treatment CA125, preoperative CA125, preoperative clinical stage, chemotherapy regimen, optimal cytoreduction status, final surgical stage, preoperative hemoglobin and number of units of packed red blood cells transfused. Women lost to follow up were censored from survival analysis. For the primary analysis of PFS and the secondary endpoint of OS, the difference in treatment arms was assessed using Kaplan-Meier (KM) methods and the log-rank test. Median, ranges, the proportion of subjects who are progression free in this cohort, hazard ratios and corresponding 95% confidence intervals (as calculated using a Cox proportional hazards regression model) are presented. In addition, differences in the frequency distributions of baseline covariates by transfusion type were compared via Chi-square or Fisher’s exact test test for categorical variables and a t-test for continuous variables. Data analysis was performed using the SAS v9.4 software. The study protocol was submitted to the UW-Madison Institutional Review Board and granted exemption status.

## Results

**Fig. 1:**
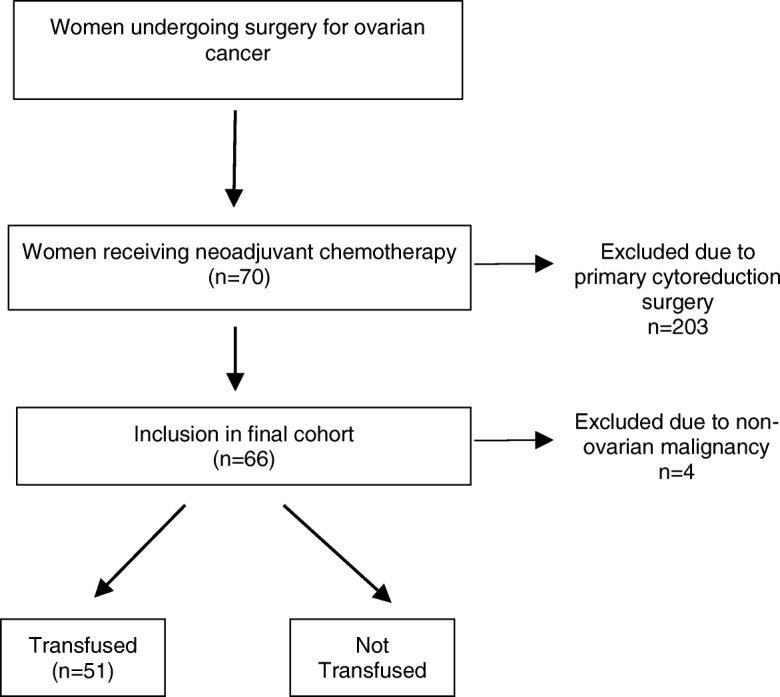
Study population: women treated for ovarian cancer at University of Wisconsin between 2010 and 2013

We identified 273 women who received surgical treatment of ovarian cancer at the University of Wisconsin between 2010 and 2013. A total of 70 (25%) women underwent NACT prior to surgical cytoreduction, as compared to 203 (75%) women undergoing primary cytoreduction surgery. Four women undergoing NACT were excluded in our final analysis; three had primary uterine cancer and one had primary appendiceal carcinoma. Sixty-six women were included in the final cohort of women who undergoing interval cytoreductive surgery after NACT (Fig. 1). The mean age of women included in the study was 69 years old, with a range from 33 to 87. The mean BMI in our cohort was 28 kg/m2, with a range from 18 to 49. An overwhelming majority of patients in the cohort had high grade papillary serous histology (91%). Other histologies included low grade serous (3%), clear cell (3%), mucinous (1.5%), and endometrioid (1.5%). The mean pre-treatment CA125 for the overall cohort was 2818 units/mL, with a range from 15 to 30,536 units/mL. The mean pre-surgery CA125 (last documented value prior to date of surgery) was 259 units/mL, with a range from 3 to 6190 units/mL. Of the 66 women included in the final cohort, 22 patients (33%) had a normal CA125 prior to surgery. Given that residual tumor burden is the most important surgical variable for ovarian cancer survival, we gathered this data on surgical outcome for the entire cohort. Optimal debulking status was known for all patients. Seventeen out of 66 (26%) had no visible disease after surgery. Twelve out of 66 (18%) had cytoreduction to 1–5 mm of visible disease. Twenty-nine of 66 (44%) of patients had cytoreduction to less than or equal to 1 cm of visible disease. Eight of 66 (12%) had a suboptimal cytoreduction. Operative blood loss was also known for all patients, with a mean intraoperative blood loss of 539 mL (range 100–2000 mL). Among all patients, the mean preoperative hemoglobin concentration was 10.6 g/dL.

The majority of our patients received a platinum-containing doublet delivered every 3 weeks prior to surgery. Fifty-five out of 66 (83%) patients received neoadjuvant carboplatin and paclitaxel. Six out of 66 (9%) received carboplatin, paclitaxel, bevacizumab prior to surgery. Five out of 66 (8%) of patients received NACT with some other chemotherapy regimen (four with carboplatin/taxotere, one with carboplatin/gemcitabine). Four women received paclitaxel on a weekly (i.e. dose-dense) schedule. The majority of women in our cohort received 3 cycles of neoadjuvant chemotherapy (66%). Forty-seven patients (71%) had clinical stage III disease at the beginning of treatment, with 17 (26%) patients having clinical stage IV disease. One patient had stage I disease; this patient presented to our facility with biopsy-proven clear cell cancer and extensive pulmonary emboli. She was managed medically with anticoagulation and neoadjuvant carboplatin-paclitaxel. Final surgical staging confirmed unilateral ovarian disease without contralateral ovarian involvement; given her preoperative biopsy she was managed as stage Ic. The median time to progression among the entire cohort was 7.7 months (range 0-53 months). The median time to death (overall survival) was 22.5 months (range 1 to 62.3 months. At the conclusion of the study, 19 patients were alive, and 45 were deceased. Two patients were lost to follow up without survival data available. Four women were in complete remission at the conclusion of the study.

**Fig. 2:**
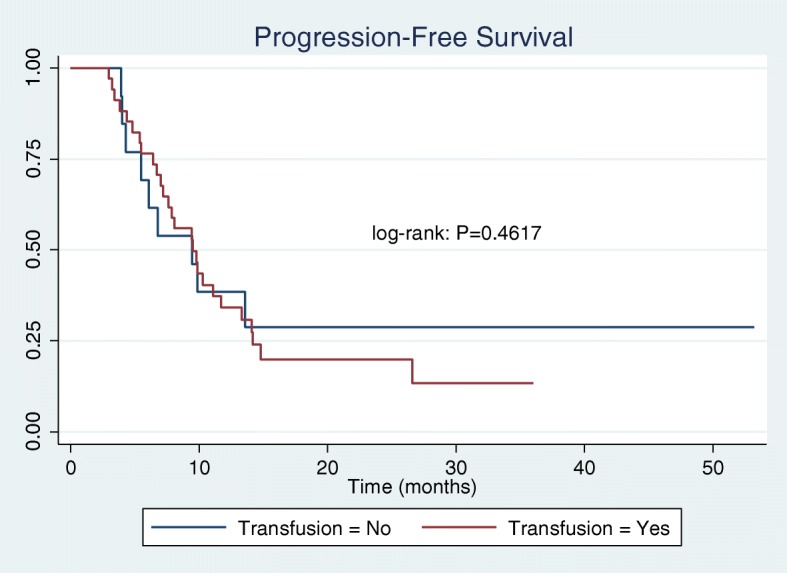
Kaplan-Meier Analysis of patients undergoing interval cytoreduction after NACT. Median time to progression was compared between patients who received perioperative blood transfusion (red line) versus those who did not (blue line). PFS = 7.6 months for transfusion group; PFS = 9.44 no transfusion group. Data were analyzed using the log-rank test

**Fig. 3:**
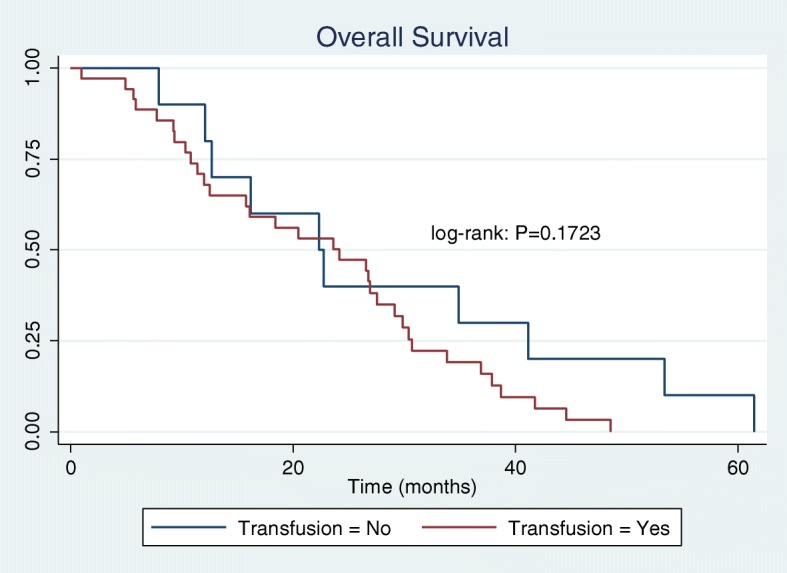
Kaplan-Meier Analysis of patients undergoing interval cytoreduction after NACT

The baseline characteristics of women in the transfusion and non-transfusion groups are shown in Table 1. A total of 51 women (77%) were exposed to allogeneic perioperative pRBC transfusion. Fifteen women (23%) were not exposed to transfusion. The mean number of units of packed red blood cells transfused was 2.98 units. The baseline characteristics were generally well-matched*.* There was no observed relationship between the number of preoperative chemotherapy cycles and the likelihood of exposure to blood transfusion. Women who were not exposed to a perioperative blood transfusion were more likely to have a normalized CA125 prior to undergoing cytoreductive surgery. (CA125 <35 units/mL, p=0.005). Preoperative hemoglobin concentration was observed to be lower in the group exposed to blood transfusion (10.5 g/dL vs 11.5 g/dL, *p*<0.009), and also observed to have a higher mean operative blood loss (613.7 mL vs 283.3 mL, *p*=0.004).The results of Kaplan-Meier analysis of our primary and secondary outcomes of interest are shown in Figs. 2 and 3. Perioperative transfusion was not associated with a significant difference in progression free survival. Among women exposed to transfusion a median PFS of 7.6 months was observed, as compared to a median PFS of 9.4 months among women not exposed to transfusion (log-rank test *p* = 0.4617). Similarly, there was no observed difference between groups for overall survival (23.6 months in transfused group, 22.5 months in not transfused group; log-rank test *p* = 0.1723). We hypothesized that an inverse dose relationship might exist between the number of units transfused and survival. Using a Cox proportional hazards model, we found no significant association between number of units transfused and OS (HR = 1.06, CI: 0.92–1.23) nor PFS (HR = 1.14, CI: 0.87–1.50). Previous studies have demonstrated a pre-surgery normal CA125 as a positive prognostic variable. In our cohort a normal CA125 trended towards an observed increase in PFS, however there was no statistical difference in OS.

**Table 1:** Baseline characteristics

Variable	Perioperative Transfusion: Yes (N=51) Mean (SD)	Perioperative Transfusion: No (N=15) Mean (SD)	*P*-value
Age	69.0 (11.46)	71.1 (9.60)	0.5278
BMI	28.0 (7.31)	26.7 (5.68)	0.5183
Histology:			
High grade pap serous	46	14	0.299
Low grade	2	0	
Mucinous	1	0	
Clear cell	2	0	
Endometrioid	0	1	
Pre-treatment CA125	3013.4 (5833.6)	2156.9 (2897.4)	0.5863
Pre-operative CA125	198.8 (401.6)	462.8 (1585.2)	0.2822
Normal Pre-op:			
Yes	11	9	0.005
No	39	6	
Delta CA125	2867.5 (5702.6)	1694.2 (1592.9)	0.4361
Pre-op Clinical Stage:			
Stage 3	36	11	0.992
Stage 4	13	4	
Pre-op Chemo:			0.907
Carboplatin/Paclitaxel	41	13	
Carbo/taxol/bevacizumab	5	1	
Other	4	1	
Dose-dense Taxol:			0.258
Yes	4	0	
No	46	15	
Pre-op Chemotherapy cycle #			0.21
3 or less	37	8	
< 3	14	7	
Optimal Cytoreduction:			0.812
No visible	12 (23.5%)	5 (33.3%)	
1-5mm	9 (17.6%)	3 (20%)	
≤1cm	22 (43.1%)	6 (40%)	
Suboptimal	7 (13.7%)	1 (6.7%)	
Final Stage:			0.646
Ic	1		
IIIc	38	10	
IVa	8	4	
IVb	4	1	
Pre-op HgB (g/dL)	10.5 (0.16)	11.5 (0.44)	0.0091
Operative blood loss (mL)	613.7 (421 )	283.3 (144.7 )	0.004
Number of Units Transfused			
zero units	0	15	
1-3 units	36	0	
4-7 units	15	0	
Days to death	662.9 (65.7)	865.8 (177.6)	0.1959
Alive:			0.918
Yes	14	4	
No	35	10	

Median time to death was compared between patients who received perioperative blood transfusion (red line) versus those who did not (blue line). OS = 23.6 months for transfusion group; OS = 22.5 months for no transfusion group. Data were analyzed using the log-rank test

## Discussion

Collectively, our experience from a single academic tertiary referral institution demonstrates a high utilization of perioperative pRBC transfusion at the time of surgery among women undergoing NACT for ovarian cancer. To our knowledge, this is the first study to report the prevalence of blood transfusion among women undergoing NACT for ovarian cancer in North America. Specifically, our cohort was collected over a time interval during which an increasing number of women are being treated with primary chemotherapy and interval cytoreduction surgery. Patients receiving blood product transfusion face risks including infectious, allergic reactions, non-allergic immune reactions, and volume overload. Data from other patient populations suggests that allogeneic blood product transfusion may be associated with poorer oncologic outcomes [3–7]. This has been demonstrated in advanced ovarian cancer patients undergoing primary cytoreduction surgery for stage III disease where transfusion was associated with a shortened median time to progression or survival of 6 months and 22 months, respectively [8]. Our cohort specifically identified women undergoing surgery after NACT for ovarian cancer because we hypothesized that this patient population is at increased risk of chronic anemia secondary to bone marrow suppression from carboplatin and paclitaxel. Interestingly, we observed no relationship between number of preoperative chemotherapy cycles and likelihood of exposure to perioperative blood transfusion. Nevertheless, in comparison to the patients included in the study of De Oliveira et al. we observed a lower preoperative hemoglobin level in our cohort (10.5 g/dL vs 11.2 g/dL) [8]. Congruently, we observed a higher rate of transfusion (77% vs 56%). We did not detect a difference in progression-free survival between women exposed to blood transfusion compared with women who were unexposed. Similarly, for the secondary outcome of overall survival, we did not observe a significant difference associated with exposure to blood transfusion. Two variables were significantly associated with exposure to transfusion: preoperative hemoglobin level and operative blood loss. While these are intuitive risk factors for transfusion, it is unlikely that operative blood loss can be reliably modified in patients undergoing cytoreductive surgery. As a result, correction of preoperative anemia may present an opportunity for future efforts geared towards reducing peri-operative blood transfusion.

Our data warrants contextualization with key articles from Vergote et al.*..* and the CHORUS trial that investigated the non-inferiority of NACT with interval cytoreduction in ovarian cancer patients [10, 11]. For our cohort, the overall median survival was 22.5 months. In comparison, Vergote et al.. observed a 30-month median overall survival in the NACT group. The CHORUS trial reported median overall survival of 24 months. It may be that the observed differences represent a relatively higher burden of disease in our patient population. Several lines of evidence support this: first, whereas Vergote et al.. observed stage IV disease in 23% of their patients, we observed stage IV disease in roughly 26% of our cohort. A similar rate of Stage IV disease was reported in the CHORUS trial (25%). Second, Vergote et al observed a pretreatment CA125 of ~ 1100 units/mL. In contrast, we observed a pretreatment CA125 that was nearly three times greater (~ 2800 units/mL). Though it was collected, the authors of the CHORUS trial did not publish the pretreatment CA125 levels in their study. In addition, Kessous et al recently published their experience from a retrospective cohort of North American women undergoing treatment for ovarian cancer and observed that women who received NACT had a roughly 3-fold higher CA125 level prior to treatment [12]. Collectively, these differences give support to the general practice patterns in the United States and North America that NACT is often reserved for patients with significant medical co-morbidities and/or a high burden of disease that would limit an optimal surgical effort. An important comparison between our cohort and the above mentioned trials is the apparent difference in the rates of blood transfusion between our U.S. cohort and the published European trials. While neither the Vergote study nor the CHORUS trial report specifically on blood product utilization, they do report grade 3 and 4 hemorrhage using Common Terminology Criteria for Adverse Events (CTCAE). Using these criteria, grade 3/4 hemorrhage would include all women undergoing blood transfusion of at least 2 units of packed red blood cells. The rate of grade 3 or 4 hemorrhage events among women undergoing NACT in these European trials ranged from 3 to 7%. In stark contrast, 77% of women undergoing NACT in our cohort received a blood transfusion. While these two outcomes are not directly comparable, our data suggests that ovarian cancer patients who are treated with primary chemotherapy in North America represent a qualitatively different patient population that is at increased risk of exposure to allogeneic blood transfusion.

Our study has several shortcomings that limit its general applicability. Our design was retrospective and indications for transfusion are not standardized among providers. Moreover,

at our institution patients are often initiated on NACT by referring medical oncologists prior to a consultation with a gynecologic oncologist. This creates multiple opportunities for unmeasured variables to alter our results and conclusions. Moreover, the small number of subjects likely limited our ability to observe differences between groups that may exist. Future studies incorporating data from multiple institutions and/or collected prospectively with standardized procedures would address some of these limitations.

## Conclusion

In conclusion, we found a high rate of perioperative allogeneic blood transfusion in women undergoing interval cytoreductive surgery for ovarian cancer after NACT. Larger and prospective trials will help further evaluate the relationship between blood transfusion and oncologic outcomes among women receiving NACT. We identified preoperative anemia and operative blood loss as risk factors for exposure to peri-operative transfusion. Future studies to evaluate the feasibility of correcting preoperative anemia while women are undergoing NACT may help reduce blood product utilization in this at-risk population.

## Additional file

Additional file 1: “Deidentified raw clinical data”. Raw clinical data, “Deidentified raw clinical data” includes all of the raw data used for analysis in the above study. (XLSX 17 kb)

## Abbreviations

CTCAE: Common Terminology Criteria for Adverse Events
NACT: Neoadjuvant chemotherapy
